# Functional Tooth Regeneration Using a Bioengineered Tooth Unit as a Mature Organ Replacement Regenerative Therapy

**DOI:** 10.1371/journal.pone.0021531

**Published:** 2011-07-12

**Authors:** Masamitsu Oshima, Mitsumasa Mizuno, Aya Imamura, Miho Ogawa, Masato Yasukawa, Hiromichi Yamazaki, Ritsuko Morita, Etsuko Ikeda, Kazuhisa Nakao, Teruko Takano-Yamamoto, Shohei Kasugai, Masahiro Saito, Takashi Tsuji

**Affiliations:** 1 Research Institute for Science and Technology, Tokyo University of Science, Noda, Chiba, Japan; 2 Division of Orthodontics and Dentofacial Orthopedics, Graduate School of Dentistry, Tohoku University, Sendai, Miyagi, Japan; 3 Department of Biological Science and Technology, Graduate School of Industrial Science and Technology, Tokyo University of Science, Noda, Chiba, Japan; 4 Organ Technologies Inc., Tokyo, Japan; 5 Oral Implantology and Regenerative Dental Medicine Graduate School, Tokyo Medical and Dental University, Bunkyo-ku, Tokyo, Japan; University of California, Merced, United States of America

## Abstract

Donor organ transplantation is currently an essential therapeutic approach to the replacement of a dysfunctional organ as a result of disease, injury or aging *in vivo*. Recent progress in the area of regenerative therapy has the potential to lead to bioengineered mature organ replacement in the future. In this proof of concept study, we here report a further development in this regard in which a bioengineered tooth unit comprising mature tooth, periodontal ligament and alveolar bone, was successfully transplanted into a properly-sized bony hole in the alveolar bone through bone integration by recipient bone remodeling in a murine transplantation model system. The bioengineered tooth unit restored enough the alveolar bone in a vertical direction into an extensive bone defect of murine lower jaw. Engrafted bioengineered tooth displayed physiological tooth functions such as mastication, periodontal ligament function for bone remodeling and responsiveness to noxious stimulations. This study thus represents a substantial advance and demonstrates the real potential for bioengineered mature organ replacement as a next generation regenerative therapy.

## Introduction

Donor organ transplantation is currently essential to replace a dysfunctional organ and to restore organ function *in vivo* [1], [2]. This approach is problematic for clinicians however as donor organs are constantly in short supply [2], [3]. An attractive new concept in current regenerative therapy that may possibly replace conventional transplantation in the future is stem cell transplantation therapy [4], [5] or a two-dimensional uniform cell sheet technique [6], [7] to repair the local sites of the damaged tissues and organs [8]. The ultimate goal of regenerative therapy in the future is to develop organ replacement regenerative therapies that will restore lost or damaged tissues following disease, injury, or aging with a fully functioning bioengineered organ [9], [10], [11]. To construct a bioengineered organ, one of two major concepts is to construct fully functional artificial organs using three-dimensional tissue-engineering technology, involving biodegradable materials and various cell types, that can immediately function after transplantation *in vivo* [12], [13], [14]. However, further technological developments are required to create such artificial organs which can immediately function [15].

For the regeneration of ectodermal organs such as a tooth, hair follicle or salivary gland [16], [17], a further concept has been proposed in which a bioengineered organ is developed from bioengineered organ germ by reproducing the developmental processes that take place during organogenesis [11], [18]. Tooth regenerative therapy is thought to be a very useful study model for organ replacement therapies [11], [19], [20]. The loss of a tooth causes fundamental problems in terms of oral functions, which are achieved in harmony with the teeth, masticatory muscles and the temporomandibular joint under the control of the central nervous system [21]. It has been anticipated that a bioengineered tooth could restore oral and physiological tooth functions [19]. We have previously developed a three-dimensional cell manipulation method, designated the organ germ method, for the reconstitution of bioengineered organ germ, such as a tooth or whisker follicle [22]. This bioengineered tooth erupted with the correct structure, occluded at the lost tooth region in an adult mouse. It also showed sufficient masticatory performance, periodontal functions for bone remodeling and the proper responsiveness to noxious stimulations [20]. This previous study thus provided a proof of concept that successful replacement of an entire and fully functioning organ could be achieved through the transplantation of bioengineered organ germ i.e. a successful organ replacement regenerative therapy [20].

Transplantation of a bioengineered mature organ will lead to immediately perform of the full functions *in vivo* and have a profound impact on the survival outcomes of many diseases [2], [9]. Transplanted bioengineered organs are also expected to be viable over the long-term and achieve the continuous production of various functional cells and their progenitors from stem cells as efficiently as the natural organ *in vivo* [23], [24]. It has also been proposed that mature organs can be developed from bioengineered organ germ by faithfully reproducing *in vivo* developmental processes. In the dental treatment, it has been expected to transplant of a bioengineered tooth unit comprising mature tooth, periodontal ligament (PDL) and alveolar bone into the tooth loss region through bone integration, which is connected between recipient bone and bioengineered alveolar bone in a bioengineered tooth unit [25]. Transplantation of a bioengineered tooth unit has also been proposed as a viable option to repair the large resorption defects in the alveolar bone after tooth loss [26]. However, there are currently no published reports describing successful transplantation or replacement using a bioengineered tooth [10], [27].

In our current study, we have generated a bioengineered tooth unit, which was controlled for length and shape and report a successful tooth replacement by transplantation of a bioengineered tooth unit into the tooth loss region, followed by successful bone integration, and restoration of tooth physiological functions such as mastication, PDL function and an appropriate responsiveness to noxious stimulations. This transplantation of a bioengineered tooth unit could also regenerate alveolar bone formation in a vertical direction. Our results thus further demonstrate the potential for bioengineered tooth replacement as a future regenerative therapy.

## Results

### Generation of a Bioengineered Tooth Unit

**Figure 1:**
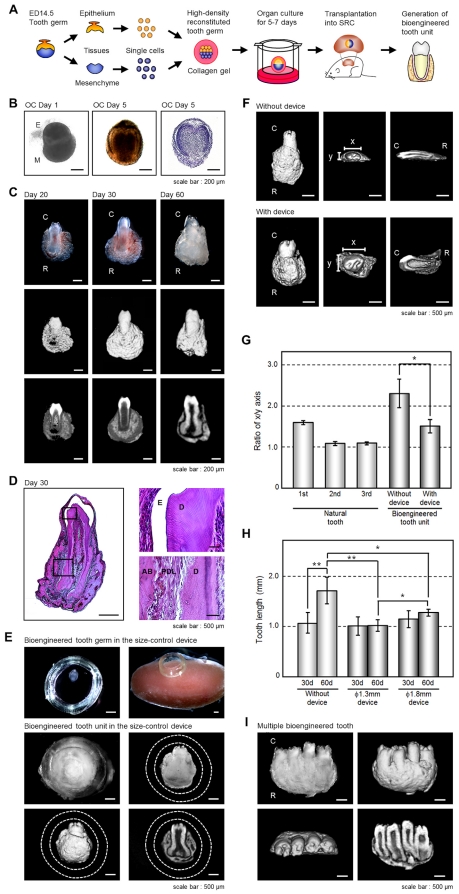
Generation of a bioengineered tooth unit. (A) Schematic representation of the generative technology of bioengineered tooth unit. (B) Phase construct imagery of a bioengineered tooth germ on day 1 (*left*) and 5 (*center*) and HE staining (*right*) of an organ culture on day 5. Scale bar, 200 µm. E, epithelium; M, mesenchyme. (C) Photographs (*upper*) and micro-CT images of the external surface area (*middle*) and cross section (*lower*) of a bioengineered tooth unit. Images were captured at 20 days (*left*), 30 days (*center*) and 60 days (*right*) after subrenal capsule transplantation (SRC). Scale bar, 200 µm. C, tooth crown side; R, tooth root side. (D) Histological analysis of the bioengineered tooth unit on day 30 after SRC transplantation (*left*). (Scale bar, 500 µm). Higher magnification images of crown area (*upper right*) and the periodontal tissue area (*lower right*) are also shown. Scale bar, 50 µm. E, enamel; D, dentin; AB, alveolar bone; PDL, periodontal ligament. (E) Photographs of the developmental processes occurring in bioengineered tooth germ in a subrenal capsule (SRC) using a size-control device. Images were captured of bioengineered tooth germ orientated in the device (*top left*), transplantation into the SRC (*top right*), and the bioengineered tooth at 50–60 days after transplantation in the SRC (middle). Micro-CT images of the external surface area (*bottom left*) and cross section (*bottom left*) are also show. The dotted lines indicate the outlines of the device. Scale bar, 500 µm. (F) Micro-CT images of a bioengineered tooth unit transplanted into the SRC for 30 days with (*lower column*) or without (*upper column*) the size-control device at an external (*left*), axial (*center*) or cross section (*right*) view. Scale bar, 500 µm. x, x-axis of the crown; y, y-axis of the crown. (G) X-axis versus y-axis ratios (x/y) of the crowns of bioengineered tooth units at 30 days post transplantation into an SRC, and also of natural first, second and third molars from 9-week-old mice. Transplantations were performed with or without the 1.3 mm thickness size-control device. Error bars show the standard deviation (*n* = 5). *P<0.001 (*t*-test). (H) The lengths of the bioengineered tooth units generated using size-control devices, which were of a 1.3 mm (ϕ1.3 mm) or 1.8 mm (ϕ1.8 mm) inner diameter, at 30 and 60 days post transplantation into an SRC were compared with or without the devices. Error bars show the standard deviation (*n* = 5). *P<0.01 and **P<0.001 (*t*-test). (I) Photograph (*first figure from the left*) and micro-CT images showing external (*second figure*), axial (*third figure*) and cross section (*fourth figure*) views of a multiple bioengineered tooth units, in which four teeth were contained in one alveolar bone, after 60 days transplantation into the SRC. Scale bar, 500 µm.

We have previously reported that bioengineered tooth germ can successfully develop a bioengineered tooth that by subrenal capsule transplantation can restore a mature tooth, including periodontal tissue and alveolar bone [22]. Because a three-dimensional *in vitro* organ culture has not yet been developed, we employed a strategy involving a bioengineered tooth unit, which has the necessary tissues to restore tooth functions, to investigation and advance the future potential of bioengineered tooth replacement (figure 1A). The bioengineered molar tooth germ was developed to a stage equivalent to the early bell stage of natural tooth germ for 5–7 days in an *in vitro* organ culture (figure 1B). Although we have previously reported that multiple bioengineered teeth have been formed from a bioengineered tooth germ reconstituted by our organ germ method [22], we recently developed a method to generate a single and width-controlled bioengineered tooth [28]. The bioengineered tooth germ gradually accumulated hard tissue, root extension, and an increased alveolar bone volume, depending on transplantation periods, and could successfully generate a tooth unit with the correct structure of a whole molar, and the proper formation of periodontal tissue and surrounding alveolar bone (figure 1C, D). However, the shape (x vs. y axis) of the bioengineered tooth unit was flattened by the pressure of the outer membrane of the subrenal capsule (figure 1F, G). The length of the tooth also showed continuous root elongation depending on the transplantation periods without occlusional mechanical stress (figure 1C, F, H).

To generate the shape- and length-controlled bioengineered tooth unit so that a suitable size was obtained for intraoral transplantation, the tooth germ was inserted into a ring-shaped size-control device and then transplanted into a subrenal capsule (figure 1E). The crown widths, calculated from the x/y axis ratios, of natural first, second and third molars of 9-week-old adult mice were 1.61±0.05 mm, 1.09±0.04 mm, 1.12±0.04 mm, respectively (each n = 5, figure 1G). The crown width of the bioengineered tooth units grown in the size-control device, which had a 1.8 mm inside diameter and 1.3 mm thickness, was 1.46±0.16 mm whereas when grown outside of the device the size was 2.30±0.35 mm (each n = 5, figure 1G). The device thus successfully generated a size-controlled bioengineered tooth so that it was similar to a natural tooth (figure 1F, G). This device could avoid the pressure by the subrenal capsule membrane, and reserve the three-dimensional space for developing a bioengineered tooth germ normally. We next evaluated the length of a bioengineered tooth unit generated in the size-control device (figure 1E). After 30 or 60 days, the lengths of the teeth transplanted without the devices were 1.07±0.20 mm and 1.70±0.26 mm, respectively, which was significantly associated with the transplantation period (each n = 5, figure 1H, figure S1A). Although the length of the bioengineered tooth transplanted without the devices was 1.70±0.26 mm after 60 days transplantation, bioengineered teeth transplanted in devices of 1.3 or 1.8 mm in diameter, was significantly regulated at 1.02±0.11 or 1.27±0.06 mm, respectively (each n = 5, figure 1H). The shape and length of the bioengineered tooth unit can therefore be controlled in three-dimensions using a specialized device.

Multiple bioengineered tooth units surrounded by alveolar bone could be also generated by the transplantation of several tooth germs into a single size-control device (figure 1I, figure S1B). Each resulting tooth had the correct structure including pulp cavities and partitioned periodontal spaces (figure 1I, figure S1C). Hence, multiple tooth replacements can be achieved with this regenerative transplantation method.

### Transplantation of a Bioengineered Tooth Unit into a Tooth Loss Region *in Vivo*

**Figure 2:**
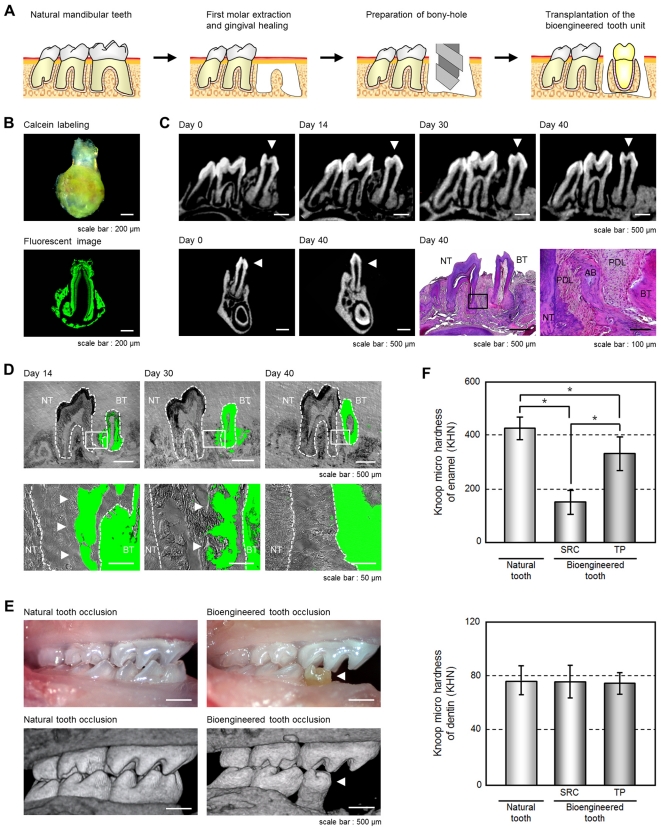
Engraftment and occlusion of a bioengineered tooth unit in a tooth loss model. (A) Schematic representation of the protocol used to transplant a bioengineered tooth unit in a murine tooth loss model. (B) Photograph (*Upper*) and sectional image (*Lower*) of a calcein-labeled bioengineered tooth unit at 60 days post transplantation in an SRC. Scale bar, 200 µm. (C) Micro-CT images of a bioengineered tooth unit (arrowhead) in cross section (*upper*) and frontal section (*first and second figures from the lower left*) during the processes of bone remodeling and connection between the recipient jaw bone and alveolar bone of the tooth unit. Histological analysis of the engrafted bioengineered tooth unit at 40 days post transplantation was also performed. (Scale bar, 500 µm and 100 µm in the lower and higher magnification figure; *third and fourth figure from the lower left*). NT, natural tooth; BT, bioengineered tooth; AB, alveolar bone; PDL, periodontal ligament. (D) Sectional images of a calcein-labeled bioengineered tooth unit at 14, 30 and 40 days post-transplantation. The calcein-labeled bone of the bioengineered tooth units (arrowhead) was found to gradually decrease from the outside and finally disappear at 40 days post-transplantation. Scale bar, 500 µm (*upper*), 50 µm (*lower*). NT, natural tooth; BT, bioengineered tooth. (E) Oral photographs (*upper*) and micro-CT (*lower*) images showing occlusion of natural (*left*) and bioengineered teeth (*right*). Scale bar, 500 µm. (F) Assessment of the hardness of a bioengineered tooth. Knoop microhardness values of the enamel (*upper*) and dentin (*lower*) of a bioengineered tooth at 60 days post-transplantation in a subrenal capsule (SRC) and at 40 days post-transplantation in jawbone (TP) were compared with those of natural teeth in 11-week-old mice. Error bars show the standard deviation (*n* = 5). *P<0.01 (*t*-test).

We next investigated whether a bioengineered tooth unit could be engrafted via the integration between the alveolar bone of this unit and that of the host recipient and then function appropriately by occlusion with an opposing tooth (figure 2A). The bioengineered tooth unit, which was generated by transplantation in a device of a 2.5 mm inside diameter for 50–60 days and labeled by the administration of calcein reagent into recipient mouse (figure 2B), was transplanted with the correct orientation into a properly-sized bony hole in the lower first molar region of the alveolar bone in a 4-week-old mouse (figure 2C). Briefly, in this mouse model, the lower first molar had been extracted, and the resulting gingival wounds had been allowed to heal for 4–6 days (figure S2A). When the bioengineered tooth unit was transplanted, it was located at a position reaching the occlusal plane with the opposing upper first molar (figure 2C, figure S2A). Partial bone integration was observed at 14 days after transplantation, and full bone integration around a bioengineered tooth root was seen at 30 days after transplantation (figure 2C). In the calcein-labeled alveolar bone of bioengineered tooth unit, resorption was partially observed at the surface at 30 days post-transplantation (figure 2D, figure S2B). The calcein-labeled bone finally disappeared and the recipient bone around the bioengineered tooth root replaced it completely at 40 days after transplantation at a frequency of 66/83 (79.5%; figure 2C, D, figure S2B). There have been many previously reported clinical cases of multiple tooth loss, the most serious condition being edentulism [29]. It is possible that a bioengineered teeth unit could be transplanted into an edentulous jaw (figure S2E, F). Our current findings suggest that bioengineered teeth can be engrafted into regions of tooth loss through bone integration, which involves resorption of the alveolar bone of the bioengineered tooth unit through natural bone remodeling in the recipient.

The engrafted bioengineered tooth was found to be aligned appropriately and occlude with the opposing upper first molar (figure 2E, figure S2C). Micro-CT analysis also revealed that no root elongation was evident for the bioengineered tooth and that the apical foramen of the engrafted bioengineered tooth root significantly narrowed at 40 days after transplantation (each n = 9, figure S2D). These results suggest that the bioengineered tooth in the tooth unit isolated from subrenal capsule transplantation is immature tooth, which has the potential to narrow of the apical foramen after the oral transplantation and would have the physiological ability to recapitulate mechanical stress by occlusion.

Masticatory potential is essential for proper tooth function and we next performed a Knoop hardness test, an important measure of masticatory functions, on bioengineered teeth including both the dentin and the enamel components. The Knoop hardness numbers (KHN) of the enamel and dentin in the natural teeth of 11-week-old adult mice were measured at 404.2±78.2 and 81.0±11.5, respectively (each n = 5, figure 2F). The bioengineered teeth generated in a subrenal capsule (SRC) and in jaw bone (TP) showed similar KHN values at 179.6±49.2 and 319.6±78.3 in the enamel, and 80.7±11.5 and 76.8±13.6 KHN in the dentin, respectively (each n = 5, figure 2F). The value of enamel Knoop hardness of natural tooth increase in according to postnatal period [20]. Although the enamel hardness of the bioengineered tooth generated in a SRC showed low KHN values, the enamel hardness of the engrafted bioengineered teeth (TP) increased to the high KHN value in according to the period after the transplantation into jaw bone. Therefore, the hardness of the dentin in the engrafted bioengineered teeth was in the normal range. These findings indicate that the hardness of the enamel and dentin in the engrafted bioengineered teeth were in the normal range.

### Functional Analysis of the Periodontal Ligament and Neurons of the Engrafted Bioengineered Teeth

**Figure 3:**
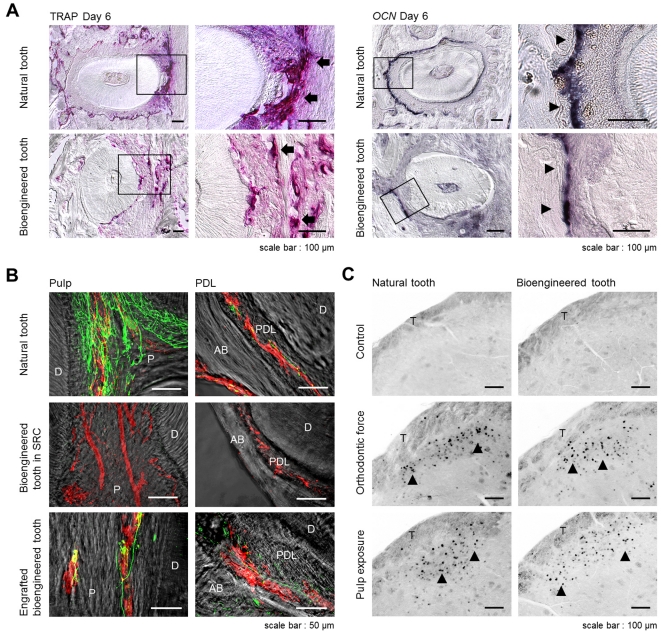
Experimental tooth movement and pain response to mechanical stress. (A) Sections of natural and bioengineered teeth were analyzed by TRAP-staining and *in situ* hybridization analysis of *Ocn* mRNA at day 6 of orthodontic treatment. TRAP-positive cells (arrow) and *Ocn* mRNA-positive cells (arrowhead) are indicated. Scale bar, 100 µm. (B) Nerve fibers and blood vessels in the pulp and PDL of a natural tooth (*top*), a bioengineered tooth unit in an SRC (*middle*), and a bioengineered tooth at 40 days after transplantation (*bottom*) were analyzed immunohistochemically using specific antibodies for neurofilament (NF; green) and von Willebrand Factor (vWF; red). Scale bar, 50 µm. D, dentin; P, pulp; AB, alveolar bone; PDL, periodontal ligament. (C) Analysis of c-Fos immunoreactive neurons in the medullary dorsal horns of mice after 0 hours (no stimulation, control; *top*), 2 hours of stimulation by orthodontic force (*middle*) and pulp exposure (*bottom*). C-Fos (arrowhead) was detectable after these stimulations in both natural (*left*) and bioengineered teeth at 40 days post-transplantation (*right*). Scale bar, 100 µm. T, spinal trigeminal tract.

Previously, it had been demonstrated that the bioengineered tooth germ can recapitulate physiological tooth function in the adult murine oral environment [20]. In our present study, we next investigated whether an engrafted bioengineered mature tooth unit can also restore physiological tooth functions *in vivo* such as the response to mechanical stress and the perceptive potential for noxious stimulations. It is essential for tooth functions that the engrafted bioengineered tooth in recipient has the cooperation with the oral and maxillofacial regions through the PDL. The response of the PDL to mechanical stress, such as orthodontic movements, induces alveolar bone remodeling, which is indicated by the localization of tartrate-resistant acid phosphatase (TRAP)-osteoclasts and osteocalcin (*Ocn*) mRNA-positive osteoblasts [20]. During experimental tooth movement, TRAP-positive osteoclasts and *Ocn* mRNA-positive osteoblasts were observed on the compression and tension sides, respectively (figure 3A). This demonstrated that the PDL of the bioengineered tooth unit successfully mediates bone remodeling via the proper localization of osteoclasts and osteoblasts in response to mechanical stress.

The perceptive potential for noxious stimulation including mechanical stress and pain, are important for proper tooth function [30]. Trigeminal ganglional neurons, which innervate the pulp and PDL, can respond to these stimulations and transduce the perceptions to the central nervous system. Blood vessels that are detected in the pulp and PDL, maintain dental tissues such as odontoblasts, pulp, the PDL and alveolar bone. In our current experiments, we evaluated the responsiveness of nerve fibers in the pulp and PDL of the engrafted bioengineered tooth to noxious stimulations. Although von Willebrand Factor (vWF)-positive blood vessels were observed in the pulp and PDL of the bioengineered tooth generated in a subrenal capsule, anti-neurofilament (NF)-immunoreactive nerve fibers could not be detected (figure 3B, figure S3A, B). However, NF-positive nerve fibers could be detected in the pulp and PDL of the engrafted bioengineered tooth in the recipient bone and the neurons merged with vWF-positive blood vessels (figure 3B). Neuropeptide Y (NPY) and calcitonin gene-related peptide (CGRP), which are synthesized in sympathetic and sensory nerves, respectively, were also detected in both the pulp and PDL neurons (figure 3B, figure S3C–F). We found in our current analyses that c-Fos immunoreactive neurons, which are detectable in the superficial layers of the medullary dorsal horn following noxious stimulations such as mechanical and chemical stimulation of the intraoral receptive fields, were present in both normal and bioengineered teeth and drastically increased in number at two hours after orthodontic treatment and pulp exposure (figure 3C). These results indicate that an engrafted bioengineered tooth unit can indeed restore the perceptive potential for noxious stimulations in cooperation with the maxillofacial region.

### Regeneration of an Extensive Bone Defect by Transplantation of a Bioengineered Tooth Unit

**Figure 4:**
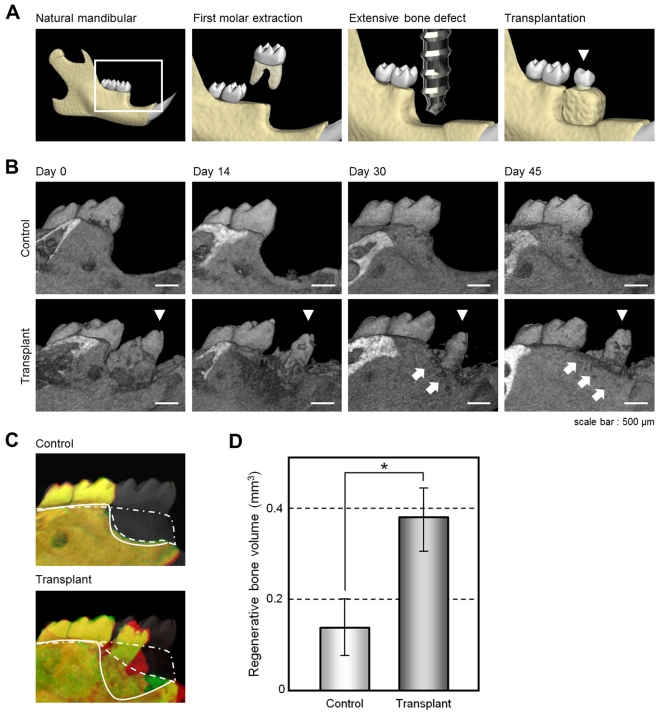
Alveolar bone regeneration following the transplantation of a bioengineered tooth unit. (A) Schematic representation of a murine extensive bone defect model and the transplantation of a bioengineered tooth unit (arrowhead). (B) Micro-CT images of the vertical alveolar bone regeneration processes in a no transplantation control (*upper*) and following the transplantation of a bioengineered tooth unit (arrowhead, *lower*) in a murine extensive bone defect model. Vertical bone formation was observed from the marginal bone of the recipient (arrow). Scale bar, 500 µm. (C) Three-dimensional superposition of micro-CT images of natural dentition (gray, double dotted line), a transplanted bioengineered tooth unit (*lower*) and a no transplantation control (*upper*) at day 0 in an extensive bone defect (red, straight line), and at 45 days after transplantation (green, dotted line). The superior edges of the recipient alveolar bone are indicated by each line. (D) Regenerative bone volume of the buccal area following the transplantation of a bioengineered tooth unit (transplant) and no transplantation (control) at day 45 in an extensive bone defect. Error bars show the standard deviation (*n* = 4). *P<0.01 (*t*-test).

Tooth loss is well known to cause significant alveolar bone resorption at the region in question [26]. Although there have been many studies of bone regenerative therapies [31], more effective methods to restore extensive bone defects during treatments such as dental implants are required and anticipated [26]. We investigated whether the transplantation of a bioengineered tooth unit would regenerate not only the missing tooth but also the surrounding alveolar bone of the recipient. To analyze whether such restoration of the alveolar bone occurred after transplantation, we developed a murine extensive bone defect model, which was prepared by the extraction of the lower first molar and then removal of the surrounding alveolar bone to generate a critical bone defect in the lower first molar region (figure 4A, figure S4A). When we transplanted a bioengineered tooth unit into this bone defect, vertical bone formation was observed from the marginal bone of the recipient at 14 days after transplantation (figure 4B, C, figure S4B). The regenerative bone volume post-transplantation significantly increased compared with a no transplant control (0.38±0.07 mm^3^ vs. 0.12±0.08 mm^3^; each n = 4, figure 4C, D), although the height and volume of the regenerated alveolar bone surrounding the bioengineered teeth was not completely recovered. These findings indicate that transplantation of a bioengineered tooth unit can restore a serious bone defect.

## Discussion

We here demonstrate the successful transplantation of a bioengineered tooth unit, which is a model for a bioengineered mature organ, into a missing tooth region *in vivo* and the subsequent restoration of tooth function by this graft. We also show that this transplantation can restore the bone volume in both the vertical and horizontal dimensions in a missing tooth mouse model with a serious extensive bone defect. These findings indicate that whole tooth regenerative therapy is feasible through the transplantation of a bioengineered mature tooth unit. This study also provides the first reported evidence of entire organ regeneration through the transplantation of a bioengineered tooth.

Organ replacement regenerative therapy, but not stem cell transplantation regenerative therapy for tissue repair, holds great promise for the future replacement of a dysfunctional organ with a bioengineered organ reconstructed using three-dimensional cell manipulation *in vitro* [11], [19]. In previous reports, however, artificial organs, which were constructed with various cells and artificial materials could not restore functionality and thus are not a viable option for long-term organ replacement *in vivo* [15]. Previously, it has been shown that a bioengineered organ can be grown *in vivo* in amphibian models in which activin-treated cell aggregates could form a secondary heart with pumping function and also regenerate eyes that were light responsive and connected with the host nervous system [32], [33]. Recently, we have also regenerated bioengineered organ germs, including tooth germs and whisker follicles, and successfully achieved a fully functioning tooth replacement in an adult mouse through the transplantation of a bioengineered tooth germ in the lost tooth region [20], [22]. It has been anticipated that replacement therapies will be developed in the future through the transplantation of a bioengineered mature organ with full functionality and long-term viability [2], [19]. In our present experiments, we successfully generated a size-controlled bioengineered mature tooth unit, a strategy we adopted because the growth of functional organs *in vitro* is not yet possible [27]. Organs require a sufficient mass (cell number) and proper shape to function [34] and the tooth has unique morphological features, such as the tooth crown width and length (macro-morphology), and cusp and root shape (micro-morphology) [35]. However, the technology to regulate tooth morphogenesis for whole tooth regeneration remains unexplored [36]. We recently developed a novel organ germ method to regulate the crown width by regulating the contact area between epithelial and mesenchymal cell layers [28]. In our previous work, we demonstrated that the length of the bioengineered tooth is equivalent to that of natural tooth after the transplantation of the bioengineered tooth germ into oral environment [20]. In this study, the length of the bioengineered tooth unit could be controlled longitudinally, which would be provided by the limited space of the device. These findings provide the first evidence that the bioengineered tooth can be controlled in three-dimensions using a specialized device. It is also thought that bioengineered teeth could be generated with a controlled crown width through cell manipulation and tooth length by placement in a size-controlling device, which places a three-dimensional spatial limitation on size [20], [28].

Loss of teeth and functional disorders in the PDL or temporomandibular joint, cause fundamental problems for oral functions, such as enunciation, mastication and occlusion, and associated health issues [21]. Although, missing teeth are traditionally restored by replacement with an artificial tooth, such as a bridge, denture or osseo-integrated dental implant, it is thought that the proper restoration of tooth functions will require bone remodeling regulated by the PDL [20] and a proper responsiveness to noxious stimulations [30]. Previous reports of autologous tooth transplantations have indicated that natural periodontal tissue on the tooth could restore the physiological tooth function, including bone remodeling [37]. We recently showed that a fully functional bioengineered tooth can be achieved through the transplantation of a bioengineered organ germ [20]. In our current study, we demonstrate the successful replacement of an entire and fully functional tooth unit *in vivo*, which restored masticatory potential, the functional responsiveness, including bone remodeling, of the periodontal tissue to mechanical stress and proper responsiveness to noxious stimulations via both peripheral sensory and sympathetic nerves. This is a significant advance for the concept of whole tooth regenerative therapy in which the transplantation of a bioengineered mature organ, and not organ germ, can replace an organ and restore its full function.

In order for a tooth to cooperate with the maxillofacial region, it is supported by the connection between the root cementum and alveolar bone through the PDL, which has essential roles in tooth support, resorption and repair of the root cementum, and the remodeling of alveolar bone [38]. Tooth loss causes a large amount of alveolar bone resorption, which is mediated by the PDL, in the vertical and horizontal dimensions, and the loss of this bone, which leads to both functional and aesthetic problems, is difficult to rectify with standard dental therapies such as dental implant and autologous tooth transplantation [26]. Although bone regeneration has been attempted for many years through the use of tissue engineering technologies, guided bone regeneration methods, autologous bone or cell transplantation, and cytokine therapies with BMPs, FGFs or PDGF, no clinical protocol for bone regeneration in the vertical and horizontal dimensions has been established yet [31]. In our present study however, we demonstrate that a bioengineered tooth unit could be engrafted and integrate via recipient bone remodeling after transplantation into an extensive bone defect. The recipient alveolar bone of the vertical dimension was observed to maintain the height of the PDL in the bioengineered tooth unit. These findings indicate that the transplantation of a bioengineered tooth has great potential for not only future whole tooth regenerative therapy but also as a treatment in clinical cases where tooth loss is accompanied by a serious alveolar bone defect.

Further studies of three-dimensional organ culture technologies *in vitro*, which can generate a fully functional bioengineered organ, and the identification of available adult tissue stem cells for the reconstitution of a bioengineered tooth germ will be required in the future to realize whole tooth regenerative therapy in the clinic.

## Materials and Methods

### Ethics Statement

All animals and experimental protocols were approved by the Tokyo University of Science Animal Care and Use Committee (Permit Number: N10018). All surgery was performed under sodium pentobarbital anesthesia, and all efforts were made to minimize suffering.

### Reconstitution of a bioengineered tooth germ from single cells

Molar tooth germs were dissected from the mandibles of ED14.5 mice. The isolation of tissues and single cell preparations from the epithelium and mesenchyme has been described previously [22]. Dissociated epithelial and mesenchymal cells were precipitated by centrifugation in a siliconized microtube and the supernatant was completely removed. The cell density of the precipitated epithelial and mesenchymal cells after the removal of the supernatants reached a concentration of 5×10^8^ cells/ml [22]. Bioengineered molar tooth germ was reconstituted using our previously described 3-dimensional cell manipulation technique, the organ germ method [22]. We used 5×10^4^ epithelial and mesenchymal cells each to generate single tooth structures. The bioengineered tooth germs were incubated for 10 min at 37°C, placed on a cell culture insert (0.4 µm pore diameter; BD, Franklin Lakes, New Jersey, USA), and then further incubated at 37°C for five days in an *in vitro* organ culture as described previously [22].

### Generation of a bioengineered tooth unit

To control the length and shape of the bioengineered tooth unit, we manufactured a plastic ring-shaped structure, which was used as a size-control device, of a 1.3, 1.8 or 2.5 mm inside diameter and 1.3 mm thickness. After five days of cultivation, the reconstituted tooth germs were placed into this spacing device which was transplanted into a subrenal capsule for 60 days using 7-week-old female mice as the hosts. The bioengineered tooth unit was then isolated from the device.

### Fluorescent calcein labeling

Calcein (Wako, Osaka, Japan) was administered daily (1.6 mg/kg) via a subcutaneous dose to the transplanted bioengineered tooth germ in the subrenal capsule. These tooth units were then transplanted into the extracted regions of a lower first molar for 14, 30 or 40 days. Non-decalcified frozen sections were then prepared and observed using an Axiovert (Carl Zeiss, Oberkochen, Germany) with AxioCAM MRc5 (Carl Zeiss).

### Transplantation

The lower first molars of 4-week-old C57BL/6 (SLC, Shizuoka, Japan) mice were extracted under deep anesthesia and the resulting gingival wounds had been allowed to heal for 4–6 days. The transplantation of a bioengineered tooth unit was allowed the procedure as described previously [20]. To generate an extensive alveolar bone defect mouse model, the whole supporting alveolar bone (1.5 mm mesiodistally, 1.2 mm buccolingually and 0.6 mm vertically) was removed using a dental engine (NSK, Tochigi, Japan) under deep anesthesia. The bioengineered tooth units were transplanted into these defects using the same procedure described above.

### Microcomputed Tomography (Micro-CT)

The heads of the mice that had received a transplanted bioengineered tooth unit and normal mice were arranged in the centric occlusal position and radiographic imaging was then performed by x-ray using a Micro-CT device (R_mCT; Rigaku, Tokyo, Japan) with exposure at 90 kV and 150 mA. Micro-CT images were captured using i-view R (Morita, Kyoto, Japan) and Imaris (Carl Zeiss).

### Histochemical analysis and immunohistochemistry

Histochemical and immunohistochemical tissue analyses were performed as described previously [20], [22].

### Hardness measurements

Polished enamel and dentin samples from bioengineered tooth units extracted at 60 days after germ transplantation into the SRC or the mandible, and also a normal tooth (9-week postnatal) were embedded in acrylic resin (n = 5 for each group). The Knoop hardness test was then performed using a Miniload Hardness Tester (HM-102; Mitutoyo, Kanagawa, Japan) equipped with a Knoop diamond tip (19BAA061; Mitutoyo). Five indentations were made on each specimen with a 10 g load for 10 sec.

### Experimental orthodontic treatments

Orthodontic treatment was performed as described previously [20]. Experimental tooth movements consisted of a horizontal orthodontic force of about 10–15 g applied continuously to the bioengineered tooth of the mice in the experimental group in a buccal direction using a dial tension gauge (Mitutoyo) for six days. In the control group, orthodontic force was applied in the buccal direction to the first molars of 7-week-old normal C57BL/6 mice in the same manner as the experimental group. Serial sections at day 6 were analyzed by TRAP staining and by *in situ* hybridization analysis for osteocalcin (*Ocn*) mRNA as previously described [20].

### Pulp exposure

A minimal pinpoint mechanical exposure of the pulp was made in the bioengineered tooth or control natural first molar of mice under anesthesia using a dental engine (NSK) supplied with dental diamond point (Shofu, Kyoto, Japan). For stimulation with cold water, ice was applied to the cavity of the tooth after pulp exposure.

### Measurement of the regenerative bone volume

To evaluate the extent of the alveolar bone recovery in our extensive bone defect mouse model, we used the Micro-CT device (Rigaku) to measure alveolar bone volume of the treated areas at 0 and 45 days after transplantation. We measured the volume of the alveolar bone in the operated region using TRI/3D-BON software (Ratoc, Osaka, Japan). The 3D region of interest (ROI) was selected in the buccal alveolar bone area which was prescribed from the medial edge of lower second molar to the distal edge of the foramen mentale. We subtracted the alveolar bone volume of the area at day 0 from the volume at day 45, and calculated the regenerated bone volume.

### Statistical analysis

Statistical significance was determined with the unpaired Student's *t*-test, analyzed using the Common Gateway Interface Program (twk, Saint John's University).

## Supporting Information

Figure S1 **A method for controlling the size of a bioengineered tooth unit.** (A) Micro-CT images of the shapes of a bioengineered tooth unit, size controlled by devices of a 1.3 or 1.8 mm inner diameter, at 30 and 60 days after transplantation in an SRC. Scale bar, 500 µm. (B) Photograph of plural bioengineered tooth germ arranged in a size controlled device. Scale bar, 500 µm. (C) Micro-CT images (*left*) and histological analysis of the multiple bioengineered tooth units on day 60 after SRC transplantation (*middle and right*). The alveolar bone between the bioengineered teeth is indicated by arrowheads (*lower left*). Scale bar, 200 µm. Higher magnification images of the periodontal tissue area (*lower middle and right*) are also shown. Scale bar, 50 µm. D, dentin; AB, alveolar bone; PDL, periodontal ligament.
(TIF)

Figure S2 **Engraftment and establishment of occlusion of a bioengineered tooth unit at the tooth loss region.** (A) Oral photographs and micro-CT images of bioengineered tooth unit transplantations into the adult mandible. Images were captured of lateral (*top*), occlusal (*middle*) and cross sections (*bottom*) views. The bioengineered tooth unit is indicated by an arrowhead. Scale bar, 500 µm. (B) Sectional images of a calcein-labeled bioengineered tooth unit at 14, 30 and 40 days after transplantation into a murine model. Fluorescent and DIC images are merged. The alveolar bone of the bioengineered tooth unit is indicated by arrowheads. Scale bar, 500 µm, *upper*; 100 µm, *lower*. NT, natural tooth; BT, bioengineered tooth. (C) Oral photographs of an engrafted bioengineered tooth in a lateral view (upper left), a 45-degree view (*lower left*), an occlusal view (*upper right*) and a fluorescent image (*lower right*). Scale bar, 500 µm. (D) Measurements of the tooth length (*left*) and apical foramen width (*right*) of a bioengineered tooth at day 0 and day 40 after transplantation. Error bars show the standard deviation (*n* = 9). *P<0.05 (*t*-test). (E) Schematic representation of the protocol for transplanting multiple bioengineered tooth units in a murine edentulous model. (F) Micro-CT images of transplanted multiple bioengineered tooth units in a murine edentulous model. Images were captured of the external surface area (*left*), sagittal section (*center*) and cross section (*right*). The bioengineered teeth are indicated by the arrowheads in *the left figure*. Scale bar, 500 µm.
(TIF)

Figure S3 **Regeneration of nerve fibers and blood vessels in the engrafted bioengineered tooth unit.** (A, B) Nerve fibers and blood vessels in the pulp (A) and PDL (B) of a natural tooth (*top*), bioengineered tooth unit in an SRC (*middle*) and bioengineered tooth at 40 days after transplantation into an oral tooth loss region (*bottom*) were analyzed immunohistochemically using specific antibodies for NF and vWF. DIC (*first columns from the left*), NF images (*second columns*), vWF images (*third columns*), and merged images (*fourth columns*) are shown. Scale bar, 50 µm. (C, D) Nerve fibers in the pulp (C) and PDL (D) of a natural tooth (*top*), bioengineered tooth unit in an SRC (*middle*) and bioengineered tooth at 40 days after transplantation (*bottom*) were analyzed immunohistochemically using specific antibodies for NF and neuropeptide Y (NPY). DIC (*first columns from the left*), NF images (*second columns*), NPY images (*third columns*), and merged images (*fourth columns*) are shown. Scale bar, 50 µm. (E, F) Nerve fibers in the pulp (E) and PDL (F) of a natural tooth (*top*), bioengineered tooth unit in an SRC (*middle*) and bioengineered tooth at 40 days after transplantation (*bottom*) were analyzed immunohistochemically using specific antibodies for NF and calcitonin gene-related peptide (CGRP). DIC (*first columns from the left*), NF images (*second columns*), CGRP images (*third columns*), and merged images (*fourth columns*) are shown. Scale bar, 50 µm.
(TIF)

Figure S4 **Alveolar bone regenerative potential of a bioengineered tooth unit.** (A) Photographs of a lateral (*left*) and occlusal (*right*) view of a natural mandibular dentition and an extensive bone defect (arrowhead). Scale bar, 500 µm. (B) Micro-CT images of the frontal section of a no transplantation control (*upper*) and a transplanted bioengineered tooth unit at day 45 in a murine extensive bone defect model (*lower*). Significant vertical bone regeneration was observed following the transplantation of a bioengineered tooth unit when compared with the no transplantation control. The regenerated alveolar bone is indicated by an arrow. Scale bar, 500 µm.
(TIF)
