# Supplementary figures and images for: Functional Tooth Regeneration Using a Bioengineered Tooth Unit as a Mature Organ Replacement Regenerative Therapy
Source: PLoS One. 2011 Jul 12;6(7):e21531. doi: 10.1371/journal.pone.0021531 (PMC3134195; doi:10.1371/journal.pone.0021531)

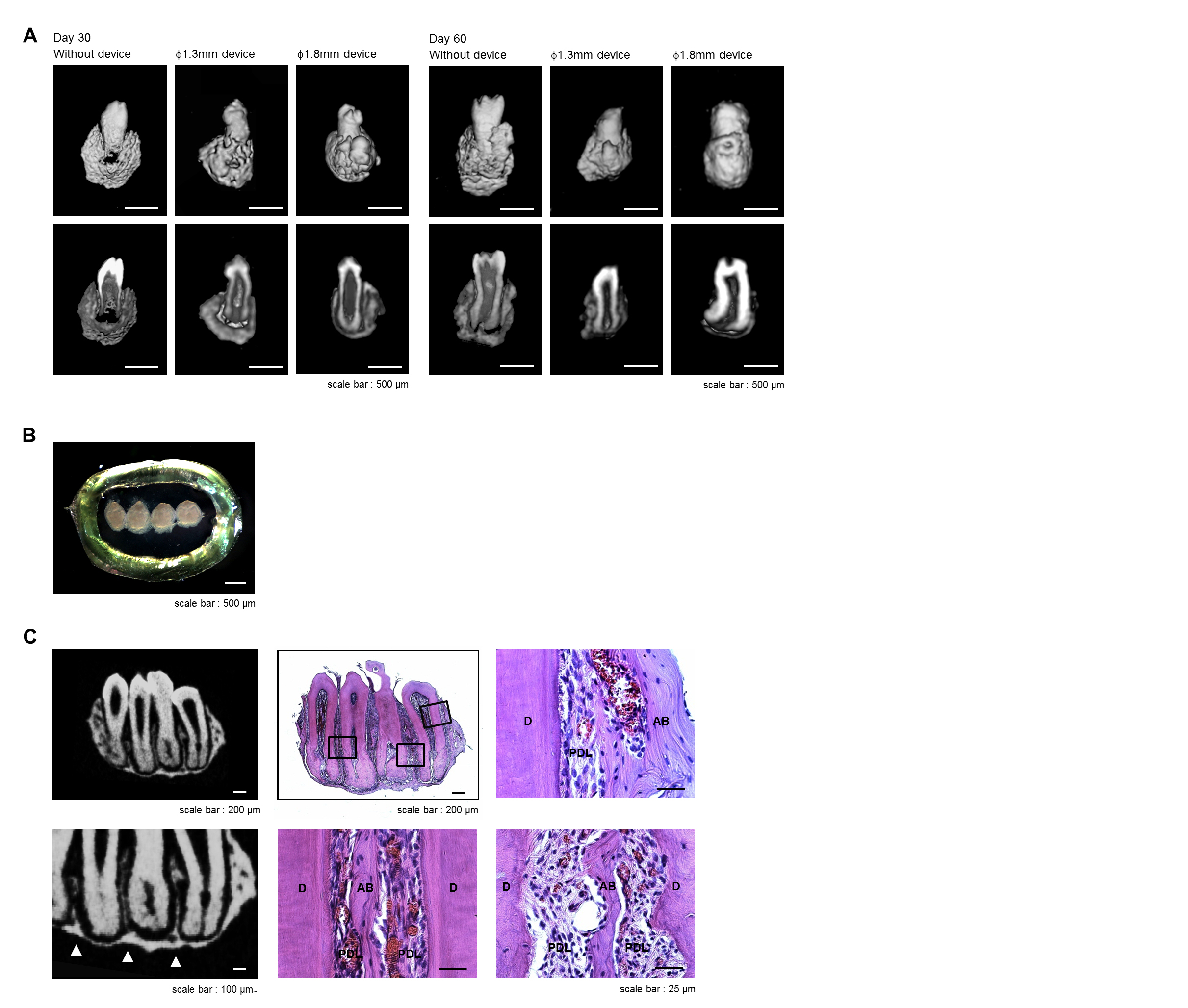

Supplement: Figure S1 — A method for controlling the size of a bioengineered tooth unit. (A) Micro-CT images of the shapes of a bioengineered tooth unit, size controlled by devices of a 1.3 or 1.8 mm inner diameter, at 30 and 60 days after transplantation in an SRC. Scale bar, 500 µm. (B) Photograph of plural bioengineered tooth germ arranged in a size controlled device. Scale bar, 500 µm. (C) Micro-CT images (left) and histological analysis of the multiple bioengineered tooth units on day 60 after SRC transplantation (middle and right). The alveolar bone between the bioengineered teeth is indicated by arrowheads (lower left). Scale bar, 200 µm. Higher magnification images of the periodontal tissue area (lower middle and right) are also shown. Scale bar, 50 µm. D, dentin; AB, alveolar bone; PDL, periodontal ligament. (TIF) [file pone.0021531.s001.tif]

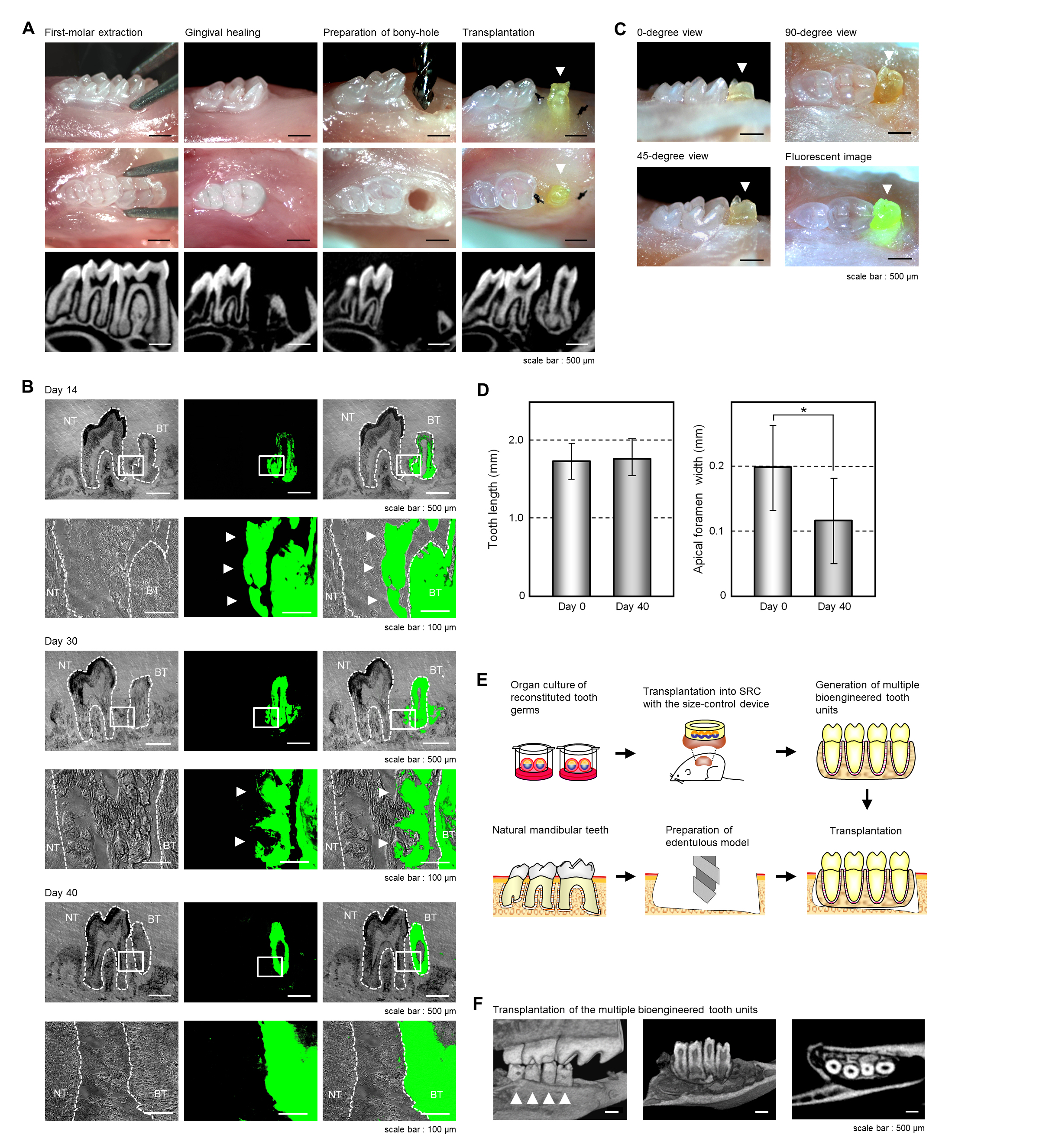

Supplement: Figure S2 — Engraftment and establishment of occlusion of a bioengineered tooth unit at the tooth loss region. (A) Oral photographs and micro-CT images of bioengineered tooth unit transplantations into the adult mandible. Images were captured of lateral (top), occlusal (middle) and cross sections (bottom) views. The bioengineered tooth unit is indicated by an arrowhead. Scale bar, 500 µm. (B) Sectional images of a calcein-labeled bioengineered tooth unit at 14, 30 and 40 days after transplantation into a murine model. Fluorescent and DIC images are merged. The alveolar bone of the bioengineered tooth unit is indicated by arrowheads. Scale bar, 500 µm, upper; 100 µm, lower. NT, natural tooth; BT, bioengineered tooth. (C) Oral photographs of an engrafted bioengineered tooth in a lateral view (upper left), a 45-degree view (lower left), an occlusal view (upper right) and a fluorescent image (lower right). Scale bar, 500 µm. (D) Measurements of the tooth length (left) and apical foramen width (right) of a bioengineered tooth at day 0 and day 40 after transplantation. Error bars show the standard deviation (n = 9). *P<0.05 (t-test). (E) Schematic representation of the protocol for transplanting multiple bioengineered tooth units in a murine edentulous model. (F) Micro-CT images of transplanted multiple bioengineered tooth units in a murine edentulous model. Images were captured of the external surface area (left), sagittal section (center) and cross section (right). The bioengineered teeth are indicated by the arrowheads in the left figure. Scale bar, 500 µm. (TIF) [file pone.0021531.s002.tif]

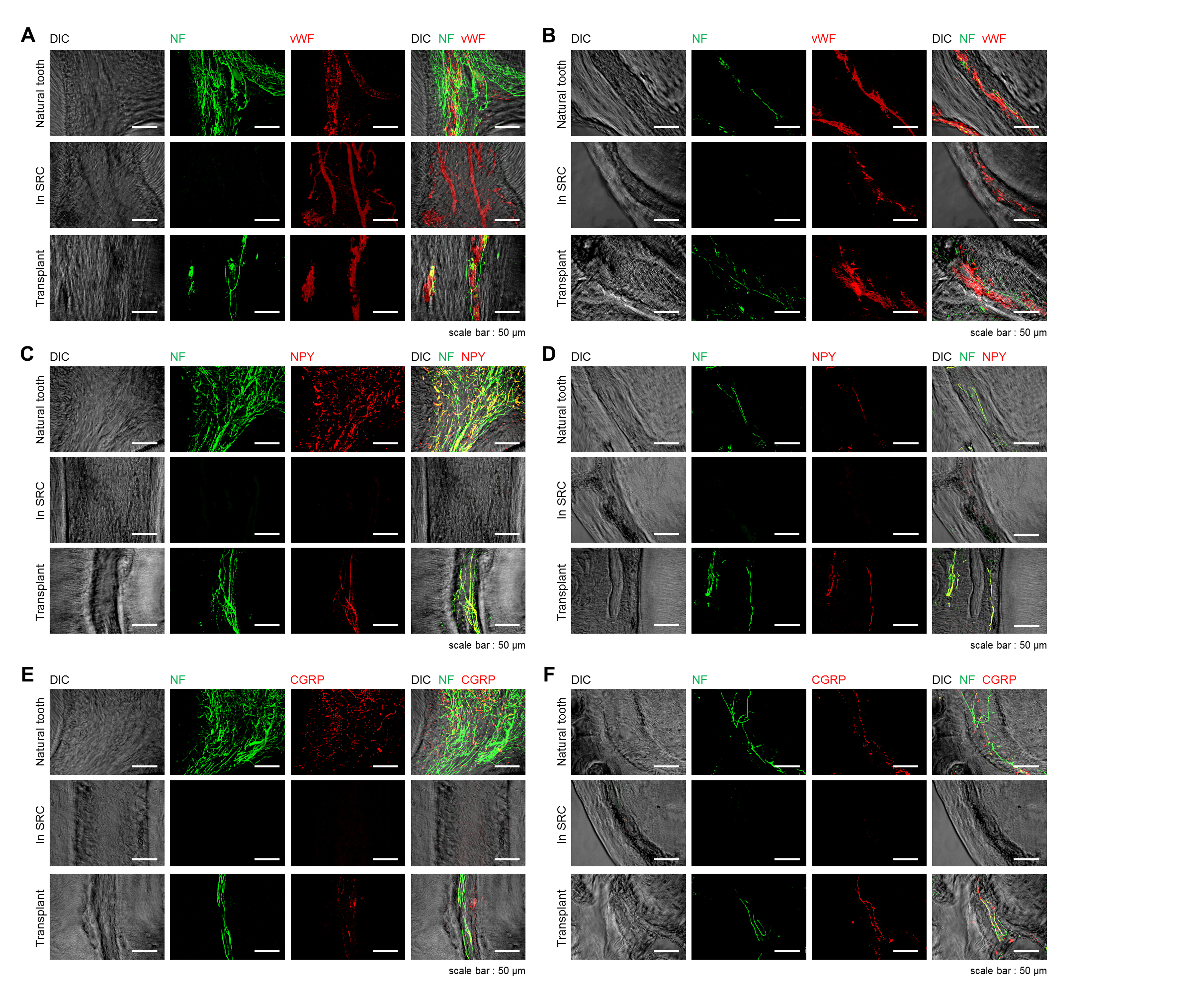

Supplement: Figure S3 — Regeneration of nerve fibers and blood vessels in the engrafted bioengineered tooth unit. (A, B) Nerve fibers and blood vessels in the pulp (A) and PDL (B) of a natural tooth (top), bioengineered tooth unit in an SRC (middle) and bioengineered tooth at 40 days after transplantation into an oral tooth loss region (bottom) were analyzed immunohistochemically using specific antibodies for NF and vWF. DIC (first columns from the left), NF images (second columns), vWF images (third columns), and merged images (fourth columns) are shown. Scale bar, 50 µm. (C, D) Nerve fibers in the pulp (C) and PDL (D) of a natural tooth (top), bioengineered tooth unit in an SRC (middle) and bioengineered tooth at 40 days after transplantation (bottom) were analyzed immunohistochemically using specific antibodies for NF and neuropeptide Y (NPY). DIC (first columns from the left), NF images (second columns), NPY images (third columns), and merged images (fourth columns) are shown. Scale bar, 50 µm. (E, F) Nerve fibers in the pulp (E) and PDL (F) of a natural tooth (top), bioengineered tooth unit in an SRC (middle) and bioengineered tooth at 40 days after transplantation (bottom) were analyzed immunohistochemically using specific antibodies for NF and calcitonin gene-related peptide (CGRP). DIC (first columns from the left), NF images (second columns), CGRP images (third columns), and merged images (fourth columns) are shown. Scale bar, 50 µm. (TIF) [file pone.0021531.s003.tif]

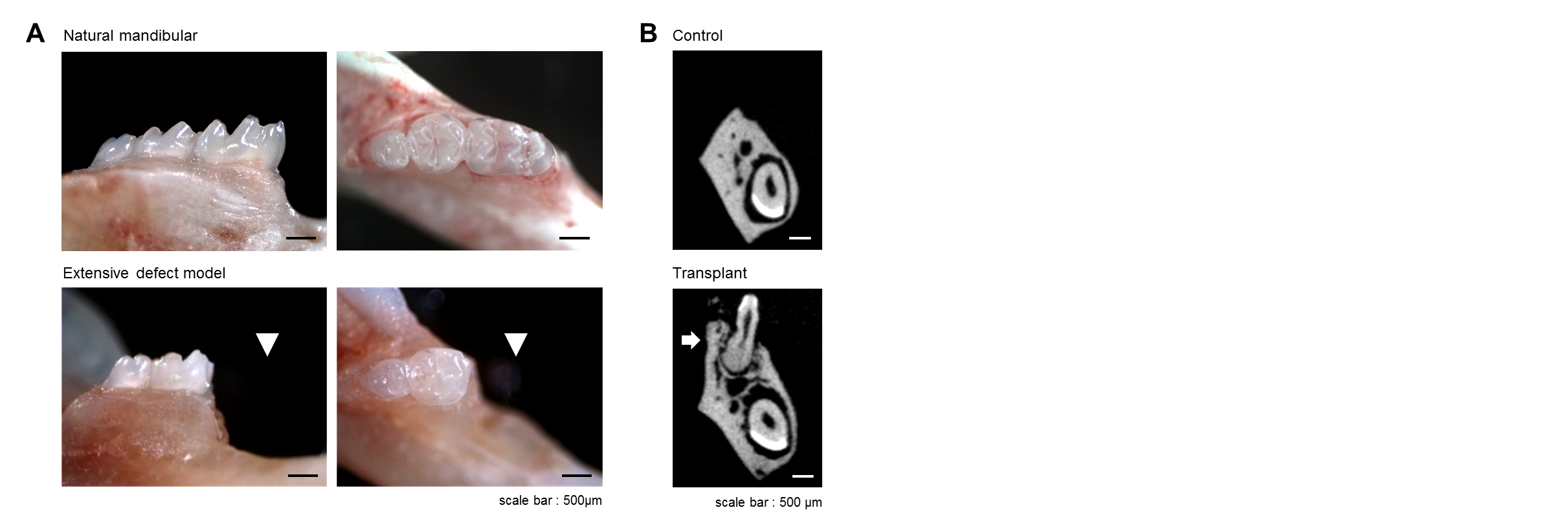

Supplement: Figure S4 — Alveolar bone regenerative potential of a bioengineered tooth unit. (A) Photographs of a lateral (left) and occlusal (right) view of a natural mandibular dentition and an extensive bone defect (arrowhead). Scale bar, 500 µm. (B) Micro-CT images of the frontal section of a no transplantation control (upper) and a transplanted bioengineered tooth unit at day 45 in a murine extensive bone defect model (lower). Significant vertical bone regeneration was observed following the transplantation of a bioengineered tooth unit when compared with the no transplantation control. The regenerated alveolar bone is indicated by an arrow. Scale bar, 500 µm. (TIF) [file pone.0021531.s004.tif]
